# Left ventricular assist device and transcatheter edge-to-edge mitral valve repair in advanced heart failure: allies or enemies?

**DOI:** 10.3389/fcvm.2023.1327927

**Published:** 2024-01-26

**Authors:** S. Valente, C. Sciaccaluga, C. Sorini Dini, F. M. Righini, M. Cameli, S. Bernazzali, M. Maccherini, V. Tarzia, G. Gerosa

**Affiliations:** ^1^ Division of Cardiology, Department of Medical Biotechnologies, University of Siena, Siena, Italy; ^2^ Department of Cardiac Surgery, University of Siena, Siena, Italy; ^3^ Cardiac Surgery Unit, Department of Cardiac, Thoracic, Vascular Sciences, and Public Health, University of Padua, Padua, Italy

**Keywords:** advanced heart failure, transcatheter edge-to-edge mitral valve repair, left ventricular assist device (LVAD), MitraClip, heart failure

## Abstract

The implantation of left ventricular assist devices (LVADs) has been increasing, with good long-term results, in parallel with a growing population with advanced heart failure (HF). However, in some European countries, LVADs are still underused, with one of the main issues being the patient's late referral. On the contrary, the use of transcatheter edge-to-edge mitral valve repair (TEER) has exponentially increased over the past decade, expanding its potential use even in patients on the heart transplantation waiting list. Even though the study populations of the main trials that investigated the prognostic impact of LVAD and TEER are different, in clinical practice a clear distinction might not be so clear. Therefore, patients with refractory HF symptoms and significant mitral regurgitation should be thoroughly evaluated through a multidisciplinary Heart Team meeting with both an advanced HF specialist and interventional cardiologist, to avoid futile procedures and to define the optimal timing for advanced HF therapies, when they are indicated. We analyzed the main available studies and registries on both TEERs and LVADs and we compared their populations and outcomes, to provide the current evidence on the use of LVAD and TEER in the HF population, especially in the light of the recently released 5-year follow-up results, giving some insights on the Italian situation, and finally to stress the importance of a solid HF network between hospitals, aiming for advanced HF patients’ timely referral for LVAD or heart transplants.

## Introduction

Heart failure (HF) is one of the major causes of morbidity and mortality (1–3) and due to advances in both diagnostic and treatment options, there is a growing portion of HF patients that eventually progresses to more advanced stages of the disease. As a matter of fact, patients with advanced HF (aHF) are estimated to represent between 1% and 10% of the overall HF population (4–6), even though defining its true prevalence remains a challenge, more so because of its evolving definitions. In fact, over the years, the effort of scientific societies was focused on the avoidance of delays in referral through an easier identification of patients with or at risk of developing aHF (7–9), and with that aim in mind, the recent mnemonic I-NEED-HELP was defined (9). The criteria for defining aHF are different, based on the adopted classification, such as the one from the Heart Failure Society of America, American College of Cardiology (ACC) and the European Society of Cardiology (ESC), with several overlaps (7, 9). In this scenario, in 2018, the Heart Failure Association of the ESC (HFA-ESC) proposed a new definition of aHF, based on four criteria, such as severe symptoms, severe cardiac dysfunction, hospitalizations/unplanned visits, and exercise impairment. According to the results of the HELP-HF registry, this classification identifies a high-risk HF population with all-cause mortality of 69.3% and HF hospitalization of 46.5% in patients fulfilling all four criteria (3). In aHF patients, all recent evidence underlines the importance of timely referral to HF centers to evaluate advanced strategies, such as heart transplants (HTx) and left ventricular assist devices (LVAD).

The HFA Atlas survey conducted in Europe between 2018 and 2019 attempted to provide data on HF epidemiology and resources for HF management among the 42 participating nations. According to the HFA Atlas, despite Italy being among the top European countries to have hospitals with dedicated HF centers, cardiopulmonary exercise testing, and HF rehabilitation programs (7.40, 4.50, and 4.50 per million people, respectively), it is one of the European nations with the lowest rate of LVAD implants (2.1 per million people) (11). Even though the data on the number of hospitals implanting LVAD in Italy were not available, it is the country with the highest number of cardiology departments performing MitraClip (1.83 per million people) treatment, followed by Germany, which however was the first European country for number of LVAD implantations (13.9 per million people) (11). However, data from ITAMACS Registry, which is the registry of Italian centers implanting mechanical circulatory support, show a growing number of LVAD implants throughout the years, in line with other European countries. In particular, between 2010 and 2021, 1,061 adult patients were supported by a long-term LVAD.

This type of information gives rise to several considerations, the first and foremost is why is the number of LVAD implants in countries such as Italy relatively low compared with other European countries while the use of the MitraClip system is fairly high? Is it possible that performing transcatheter edge-to-edge mitral valve repair (TEER) could be responsible for delaying or worst for preventing aHF patients from timely and appropriate referral for HTx/LVAD? How many patients that undergo TEER can actually be classified as aHF patients?

To answer these questions, it is essential to closely analyze the three most relevant trials related to the matter, which are the MOMENTUM 3 (Multicenter Study of MagLev Technology in Patients Undergoing MCS Therapy with HeartMate 3) trial, MITRA-FR trial (Percutaneous Repair with the MitraClip Device for Severe Functional/Secondary Mitral Regurgitation), and the COAPT (Cardiovascular Outcomes Assessment of the MitraClip Percutaneous Therapy for Heart Failure Patients with Functional Mitral Regurgitation) trial, including the recently published extended results from 5-year follow-ups from both the MOMENTUM 3 and COAPT trials (12–16).

## Comparison between study populations

Both the MOMENTUM 3 investigation and COAPT trial centered their focus on HF patients with refractory symptoms despite guideline-directed medical therapy (GDMT), even though several differences must be drawn. Tables 1, 2 summarize the inclusion criteria and the study populations of the two studies, respectively.

**Table 1:** Comparison of inclusion criteria between the MOMENTUM 3 investigation, the COAPT study, and the MITRA-FR trial.

MOMENTUM 3 trial (2016) (12)	COAPT trial (2018) (14)	MITRA-FR trial (2018) (16)
Age > 18 years	Age > 18 years	Age > 18 years
NYHA III or IV	NHYA II, III, or IV ambulatory	NHYA II, III, or IV
• Inotrope-dependent OR • CI < 2.2 L/min/m^2^, without inotropes but one criteria: - Unresponded to OMT for HF performed for at least 45 of the last 60 days - Advanced HF for at least 14 days and IABP dependent	Symptomatic secondary MR (≥3+) with • Adequately treated per applicable standards, including for coronary artery disease, LV dysfunction, MR, and HF • Subject had at least 1 hospitalization for HF in the 12 months prior to subject registration and/or a corrected BNP ≥300 pg/ml or a corrected NT-proBNP ≥1,500 pg/ml	Symptomatic severe secondary MR with • Optimal standard of care therapy for HF according to investigator • Minimum of one hospitalization for HF within 12 months preceding randomization
• LVEF ≤ 25%	• LVEF 20%–50% • LVESD ≤ 70 mm • The primary regurgitant jet is non-commissural and suitable for MitraClip implantation	• LVEF 15%–40% • EROA > 20 mm^2^ • Regurgitant volume > 30 ml
BSA ≥ 1.2 m^2^	Comorbidities preclude surgery intervention	Non-eligibility for a mitral surgery intervention according to the Heart Team
Females of childbearing age must agree to use adequate contraception	Transseptal catheterization and femoral vein access feasible	No pregnancy
Signed informed consent form	Signed informed consent form	Signed informed consent form

NYHA, New York Heart Association; LVEF, left ventricular ejection fraction; EROA, effective regurgitant orifice area; BNP, B-type natriuretic peptide; BSA, body surface area; CI, cardiac index; LV, left ventricle; LVESD, left ventricular end-systolic diameter; MCS, mechanical circulatory support; MR, mitral regurgitation.

**Table 2:** Comparison of study populations between MOMENTUM 3 investigation, COAPT study, and the MITRA-FR trial.

	MOMENTUM 3 trial (2016) (12)	COAPT trial (2018) (14)	MITRA-FR trial (2018) (16)
Age (years)	60.3 ± 12.3	71.7 ± 11.8	71.7 ± 11.8
Male (%)	79.6	66.6	78.9%
NYHA IV/INTERMACS 1-2-3-4 (%)	98.7	6.0	9.2%
LVEF (%)	17.1 ± 5.0	31.3 ± 9.1	33.3 ± 6.5
MR moderate-to-severe (%)	42.1	100.0	100
Hospitalization in the prior 12 months (%)		58.1	100
Medical therapy			
Inotropic agents (%)	86.8	0	/
ACE-inhibitors/ARNI (%)	30.9	71.5	82.2
Beta-blockers (%)	59.9	91.1	88.2
Diuretics (%)	88.2	89.4	99.3
MRA (%)		50.7	56.6
ICD (%)	66.4	30.1	31.8
CRT (%)	38.8	38.1	30.5
GFR, mean (ml/min)	60.5 ± 24.1	50.9 ± 28.5	48.8 ± 19.7
NT-proBNP (pg/ml)	//	5,174.3 ± 6,566.6	3,407 (1,948–6,790)

NYHA, New York Heart Association; INTERMACS, Interagency Registry for Mechanically Assisted Circulatory Support; LVEF, left ventricular ejection fraction; MR, mitral regurgitation; ACE, angiotensin converting enzyme; ARNI, angiotensin receptor neprilysin inhibitor; MRA, mineralocorticoid receptor antagonist, NT-proBNP, N-terminal pro b type natriuretic peptide; CRT, cardiac resynchronization therapy; GFR, glomerular filtration rate; ICD, implantable cardiac defibrillator.

The MOMENTUM 3 investigation is a prospective, multicenter, randomized pivotal trial that aimed to evaluate the safety and effectiveness of the HeartMate3 (HM3) LVAD compared with the HeartMate II LVAD in patients with advanced and refractory left ventricular HF (17). The study population was based on HF patients with the New York Heart Association (NYHA) classification III or IV, left ventricular ejection fraction (LVEF) below 25%, severe cardiac index reduction, or inotrope- or intra-aortic balloon pump-dependency (IABP) (17). Regarding the patients’ characteristics in the HeartMate 3 group, patients presented a mean LVEF of 17.1 ± 5.3% and more than half were defined as INTERMACS 3 profile (12), identifying HF patients with an advanced stage of the disease.

The MITRA-FR trial was a multicenter, randomized, open-label trial that aimed to evaluate the clinical efficacy and safety of TEER on top of optimal medical therapy (OMT) in HF patients and secondary mitral regurgitation (SMR) (16). The final study population comprised 307 patients symptomatic for HF, ranging from NYHA functional class II to IV, with severe SMR and LVEF between 15% and 40%. Each of the enrolled patients had to be hospitalized at least one time within the 12 months prior to the randomization.

The COAPT trial is a prospective, randomized and multicenter study that aimed to assess the safety and effectiveness of the MitraClip for treatment significant SMR in symptomatic HF patients despite both medical and device therapy when appropriate, such as resynchronization therapy and myocardial revascularization (18). The study population was based on HF patients with moderate-to-severe and severe SMR, range of LVEF between 20% and 50%, a NYHA functional class between II and IV ambulatory, and a previous HF hospitalization or elevated natriuretic peptides. However, among the exclusion criteria, besides moderate or severe right ventricular dysfunction and irreversible severe pulmonary hypertension, ACC/AHA stage D HF was stated. ACC/AHA stage D HF identifies patients with refractory HF symptoms despite maximum tolerated GMDT or device therapy, with frequent hospitalizations and exercise intolerance (19–21). Therefore, a clear distinction between these patients and the ones finally enrolled in the COAPT trial and MITRA-FR trial could have been challenging, and this issue is further augmented in clinical practice. In fact, on analyzing the patients’ characteristics in the device group at baseline, it was noted that 82.2% had an LVEF below 40%, 58% had experienced a HF-related hospitalization within the previous year, 54% were in NYHA class III, and the mean level of N-terminal pro-B-type natriuretic peptide (NT-proBNP) was 5,174.3 ± 6,566.6 pg/ml (18). In the intervention group of MITRA-FR trial, all patients had an LVEF below 40% and had at least one hospitalization for HF in the previous 12 months, as stated in the inclusion criteria, and 53.9% and 9.2% of them were NYHA class III and IV, respectively (16). Based on these data, it is evident that a fairly high proportion of the patients enrolled in these two investigations might fit the definition of aHF.

The EXPAND study (The MitraClip EXPAND Study of the Next Generation of MitraClip Devices) was a prospective, multicenter observational study that aimed to evaluate outcomes with the third-generation MitraClip NTR or XTR devices in patients with primary MR or SMR (22). Out of 1,041 enrolled patients, 413 had SMR, among whom 83.1% presented with NYHA class III/IV symptoms and 64.8% had at least one HF hospitalization in the previous year with the mean LVEF of 39.4 ± 13.5%.

The multicenter observational MitraBridge Registry tried to enroll a population with advanced stages of HF, including all patients with aHF, defined as NYHA class III or IV and/or with LVEF below 30%, with moderate-to-severe and severe SMR that were potential HTx candidates (23). Among the overall population of 119 patients, at the time of the MitraClip procedure, 54 patients (45.5%) were waiting for the final decision to be listed, 34 patients (28.5%) could not be listed yet (bridge to candidacy group), and finally 31 patients (26%) were on the active HTx list (23). On analysis of the patients’ characteristics, in the overall study population, it was found that 61.5% reported at least one HF hospitalization in the previous 6 months, 43.5% of the them were defined as INTERMACS profiles 5–6, and 37% as INTERMACS profiles 3–4. Compared with the COAPT study, the patients included in the MitraBridge Registry did not have to respect the echocardiographic COAPT criteria to undergo the procedure.

## MitraClip vs. LVAD: comparison between outcomes

The MOMENTUM 3 investigation showed that the centrifugal-flow HM3 LVAD was superior to the axial-flow HeartMate II LVAD with respect to survival free of disabling stroke or reoperation to replace or remove a malfunctioning device (12). Among the total study population of 1,020 patients, 515 patients were implanted with HM3 LVAD and 88.4% of them were alive after 2 years. In addition, 76.9% of them remained alive and free of disabling stroke or reoperation to replace or remove a malfunctioning device at 2 years follow-up. An excellent 2-year survival rate after HeartMate 3 LVAD implant was also confirmed by the ELEVATE Registry, which was a prospective, observational, and multicenter registry that included 540 patients, of which 463 patients received the HM3 as primary implant (24). At the 2-year follow-up, the overall survival rate of patients who received the HM3 as primary implant was 83.4% (24). This real-world population registry confirmed a significant improvement in both functional capacity and quality of life, which was sustained through the duration of the study (24). It finally confirmed the relatively low incidence of adverse events such as strokes (10.2%) and pump thrombosis (1.5%) (24). In 2021, Mehra et al. published the results of long-term outcomes in the MOMENTUM 3 pivotal trial and the continued access protocol (CAP) study phase, which overall enrolled 2,200 patients implanted with the HM3 LVAD (515 pivotal trial and 1,685 CAP) (25). The 2-year Kaplan–Meier estimates of survival free of disabling stroke or reoperation to replace or remove a malfunctioning pump were similar, attested at 76.7% in the CAP and 74.8% in the pivotal trial. The overall 2-year survival was 79% in the pivotal trial cohort and 81.2% in the CAP cohort, despite a sicker population among the latter. Finally, they reported that the survival rate of ineligible HTx patients was comparable with the one reported after HTx (25).

The investigators of MOMENTUM 3 recently published the results of the extended 5-year follow-up of the study. At the 5-year follow-up, 141 of the 515 patients (27.4%) who received the centrifugal-flow pump remained receiving LVAD support and a total of 156 underwent HTx. The extended 5-year follow-up results attested an overall Kaplan–Meier survival in the centrifugal-flow group of 58.4% (13). This overall survival rate was confirmed also in a *post hoc* analysis in the destination therapy-subgroup with centrifugal-flow pump (13). Furthermore, the 5-year Kaplan–Meier estimate of survival to transplant, recovery, or LVAD support free of debilitating stroke or reoperation to replace the pump was 54% in the HM3 group (13). Regarding cause mortality in this group, the leading causes for adverse events and deaths were right HF and infection (13).

In Italy, LVAD survival rates come from the ITAMACS Registry, attesting a 1-year survival rate of 73.5% whereas the 5-year survival rate was of 42.1%. However, it has to be underlined that these data come from a broad period of time, from 2010 to 2021, and only 34.2% of the patients were implanted with HM3, whereas 38% with HVAD, 14.2% with HeartMate II, and 13.3% with JARVIK 2000. It could be useful to further stratify survival rates according to LVAD type and time of implant, to have data that could be more easily compared with the American ones. In fact, according to the recent European PCHF-VAD Registry, the LVAD survival rate significantly increased between the years 2013 and 2020 compared with the period of 2006–2012, even though the candidate patients are much older and with multiple comorbidities (26). These data can be interpreted in light of better expertise, better patient selection, as well as improved technologies.

The COAPT Study showed that TEER using the MitraClip device in HF patients with refractory symptoms and moderate-to-severe or severe SMR improved the 2-year survival rate and reduced HF hospitalization rate compared with medical therapy alone (14). Analyzing the device group, the 2-year survival rate was 70.9%. In fact, after 2 years from the procedure, 80 patients out of 302 (29.1%) died from any cause, among which 61 patients (23.5%) died because of cardiovascular causes. The annualized rate of HF hospitalization within 2 years was 35.8% in the device group. Furthermore, 9 patients (4.4%) in the device group underwent HTx or LVAD implant compared with 22 patients (9.5%) in the control group (14).

The outcome results from a 5-year follow-up were recently published by the COAPT Investigators, confirming the superiority of MitraClip compared with medical therapy alone in reducing HF hospitalization and all-cause mortality (15). In particular, in the device group, the 5-year survival rate was 42.7% whereas the annualized rate of HF hospitalization through 5 years was 33.1% (15).

The EXPAND study showed a significant improvement in both NYHA functional class and quality of life as well as reduced HF hospitalization after TEER in both primary MR and SMR (22). However, even though the 1-year mortality rate for primary MR was significantly lower compared with the previous studies, it remained above 17% for SMR, comparable to COAPT (22). Furthermore, in NYHA class IV patients, the 1-year mortality rate was higher, around 29% (27).

The 1-year outcomes from the MitraBridge Registry were recently published, attesting that the 1-year Kaplan–Meier estimates of freedom from composite of all-cause death, urgent HTx, or LVAD implantation and first HF rehospitalization was 64% (23). Mortality rate after the procedure was 11%, mainly due to cardiac causes (HF and sudden death), urgent HTx was necessary in 7 patients (6%), and LVAD implantation, in 21 patients (18%). Elective HTx was performed in 17 patients (15%) and 23.5% no longer needed to be on the HTx list due to clinical improvement.

Conversely, in contrast to the results of the COAPT trial, the MITRA-FR investigation showed no reduction in both all-cause mortality and HF hospitalizations with TEER on top of OMT vs. OMT alone (16). The 1-year survival rate in the device group was 76%, cardiovascular death being the main determinant (21.7% of all deaths). Furthermore, almost half of the patients experienced at least one HF hospitalization after 1 year (16).

Analyzing closely the intervention groups and the control groups of both the MITRA-FR and COAPT trials, several considerations should be made. First of all, a higher proportion of the overall population of the MITRA-FR trial was receiving ACEi/ARB or angiotensin receptor-neprilysin inhibitors (ARNI) compared with the overall population of the COAPT trial (83.9% vs. 67.10%, respectively), while a similar proportion was on beta-blockers. Furthermore, patients in the medical group of both trials significantly differed with regards to ACE-ARNI use, with 85.5% in MITRA-FR vs. 62.8% in COAPT (14, 16). Although these data cannot be directly compared, this aspect might have a significant impact on the overall survival, considering the worldwide-proven benefit of these medications, limiting the benefit of TEER in the MITRA-FR trial. Even though in the COAPT trial medical therapy needed to be titrated to maximally tolerated doses, this was not specifically required in the MITRA-FR investigation and no data are available on the dosage of the drugs used in both trials.

The concept of proportionate MR vs. disproportionate MR was formulated as a possible explanation for the discordant results of these investigations (28). In fact, in the MITRA-FR trial, patients had greater left ventricular dimensions compared with the degree of MR, which defines the concept of proportionate MR in contrast to COAPT patients in whom a less severe left ventricular dilatation was found compared with more severe MR (14, 16).

## Discussion

The proportion of aHF patients has been steadily increasing due to the implementation of evidence-based therapies and consequent prolonged survival. Therefore, HF in its advanced stages has become a relevant socio-economic matter since it is associated with a high morbidity and mortality rate (2). The only two available therapies that are able to offer a significantly improved quality of life as well as higher survival are HTx and LVADs. The rate of LVAD implants in the last few decades has risen both due to the improvement of the safety profile of the devices and due to heart donor shortage, which is making HTx less and less available despite a growing aHF population. Based on the results of the analyzed studies, HF patients with SMR still have high mortality rates, especially when the burden of symptoms increases. The therapeutic goal should be to reduce this mortality rate as well as ameliorate symptoms and quality of life. In this population, TEER has been proven to significantly reduce NYHA functional class, even according to the latest trials, compared with OMT (27), while the mortality rate still remains quite high (22, 27).

**Figure 1:**
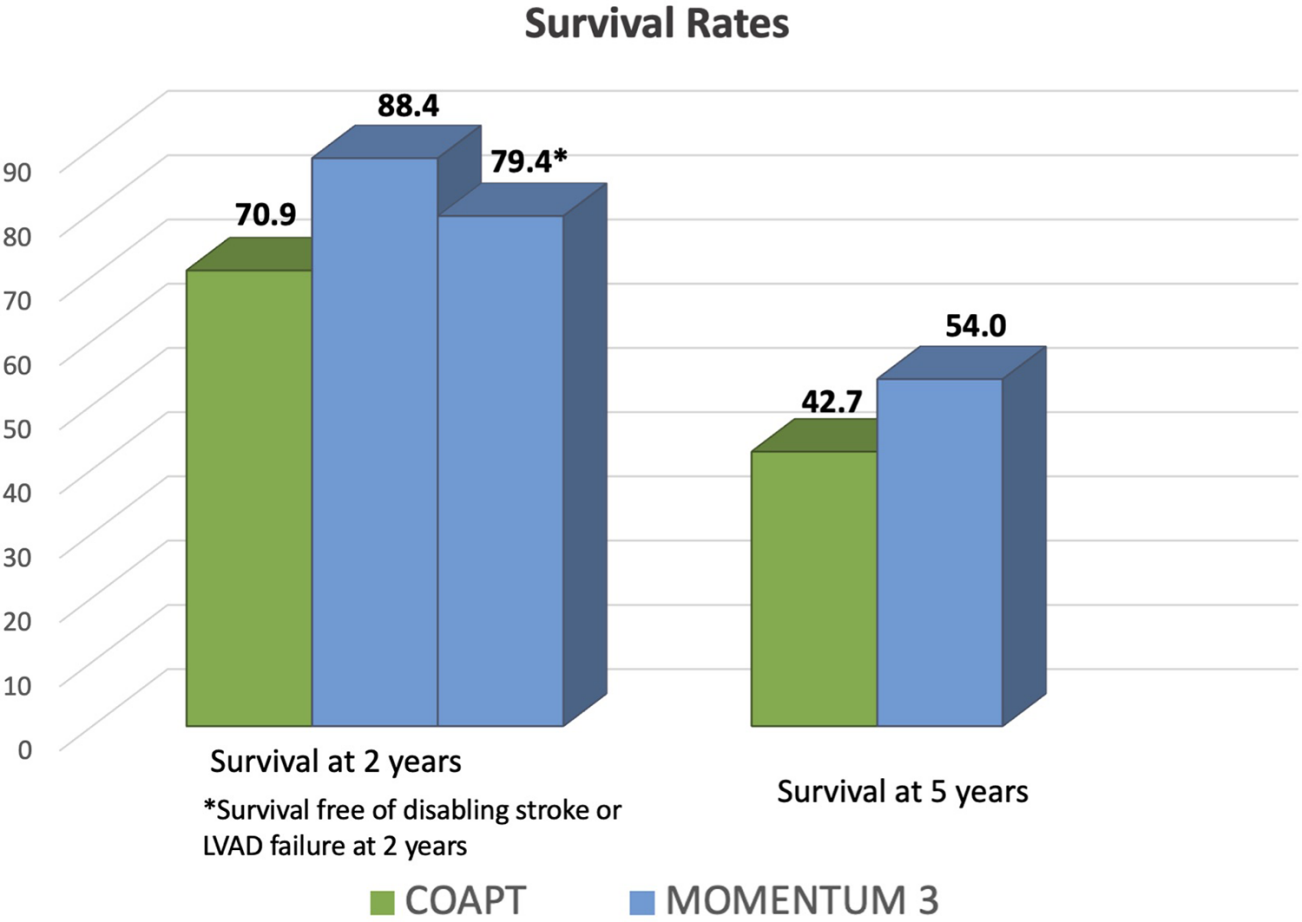
Comparison of survival rates after left ventricular assist device implant in MOMENTUM 3 investigation and after transcatheter edge-to-edge mitral valve repair in the COAPT trial. During a 2- and 5-year follow-up after LVAD implant in the MOMENTUM 3 investigation, the survival rates were 88.4% and 54%, respectively (12, 13), which is higher compared with the one from the COAPT trial. In fact, in the latter trial, the 2- and 5-year survival rates were 70.9% and 42.7%, respectively (14, 15).

As mentioned previously, the overall 2-year survival rate after centrifugal-flow LVAD is proven to be excellent, attested at 81.2% and a 2-year survival free of serious adverse event at 76.7% (25). Even though the survival rate decreases, as expected, during a 5-year follow-up post-LVAD, it still remains above 50% (13). Conversely, the 2- and 5-year survival rate after MitraClip was 70.9% and 42.7%, respectively, according to the results of the COAPT study (14, 15). Based on these results, despite a sicker population enrolled in the MOMENTUM 3 trial, the survival rate after LVAD was higher if compared with the one after the MitraClip procedure (Figure 1).

For instance, despite this evidence, Italy still remains one of the nations with the fewest implants in Europe while the use of TEER with MitraClip system has widely spread in recent years. In particular, in 2021 a total of 103 LVADs were implanted in Italy (96 of which were HM3) while in 2022 the total number of LVAD implants was 126 (29). Regarding the use of TEER in Italy, 1,157 and 1,243 procedures were performed, respectively, in 2021 and 2022 (30). Table 3 summarizes the current indication for TEER and LVAD implant according to the latest European and American guidelines.

**Table 3:** Current indication for transcatheter edge-to-edge mitral valve repair and durable left ventricular assist device.

Transcatheter edge-to-edge mitral valve repair	
ESC guidelines VHD 2021 (31)	AHA/ACC guidelines VHD 2020 (32)
• In symptomatic patients, who are judged not appropriate for surgery by the Heart Team on the basis of their individual characteristics, PCI (and/or TAVI) possibly followed by TEER (in case of persisting severe secondary MR) should be considered. (class: IIa Level: C) • TEER should be considered in selected symptomatic patients, not eligible for surgery and fulfilling criteria^a^ suggesting an increased chance of responding to the treatment. (class: IIa Level: B) • In high-risk symptomatic patients not eligible for surgery and not fulfilling the criteria suggesting an increased chance of responding to TEER, the Heart Team may consider in selected cases a TEER procedure or other transcatheter valve therapy if applicable, after careful evaluation for ventricular assist device or heart transplant. (class IIb Level C)	• In patients with chronic severe secondary MR related to LV systolic dysfunction (LVEF < 50%) who have persistent symptoms (NYHA class II, III, or IV) while on optimal GDMT for HF (stage d), TEER is reasonable in patients with appropriate anatomy as defined on TEE and with LVEF between 20% and 50%, LVESD ≤ 70 mm, and pulmonary artery systolic pressure ≤ 70 mmHg. (class 2a Level B-R)
Durable left ventricular assist device	
ESC guidelines HF 2021 (2)	AHA/ACC guidelines HF 2022 (21)
• Long-term MCS should be considered in patients with advanced HFrEF despite optimal medical and device therapy, not being eligible for heart transplantation or other surgical options, and without severe right ventricular dysfunction, to reduce the risk of death and improve symptoms. (class: IIa Level: A) • Long-term MCS should be considered in patients with advanced HFrEF refractory to optimal medical and device therapy, as a bridge to cardiac transplantation to improve symptoms, reduce the risk of HF hospitalization, and the risk of premature death. (class: IIa Level: A)	• In select patients with advanced HFrEF with NYHA class IV symptoms who are deemed to be dependent on continuous intravenous inotropes or temporary MCS, durable LVAD implantation is effective to improve functional status, quality of life, and survival. (class: 1 Level: A) • In select patients with advanced HFrEF with NYHA class IV symptoms despite GDMT, durable MCS can be beneficial to improve symptoms, improve functional class, and reduce mortality. (class 2a Level: B-R)

AHA/ACC, American Heart Association/American College of Cardiology; ESC, European Society of Cardiology; HFrEF, heart failure with reduced ejection fraction; LVESD, left ventricular end-systolic diameter; MCS, mechanical circulatory support; MR, mitral regurgitation; PCI, percutaneous coronary intervention; TAVI, transcatheter aortic valve implantation; TEE, transesophageal echocardiography; VHD, valvular heart disease.

^a^ LVEF 20%–50%, LVESD < 70 mm, systolic pulmonary pressure < 70 mmHg, absence of moderate or severe right ventricular dysfunction or severe tricuspid regurgitation, absence of hemodynamic instability.

Starting from this analysis in Italy, which could apply to other European countries, several considerations must be made. A first possible explanation could be the significant difference in the costs of these two devices, being higher for LVADs. On this matter, Baron et al. recently published a cost-effectiveness analysis of the use of the MitraClip device in the US, which showed higher cumulative 2 years costs for the MitraClip group ($73,416 vs. $38,345; *p* < 0.001), due to cost of index procedure, but showed advantages in term of increase of life expectancy by 1.13 years and quality-adjusted life-years by 0.82 years (33). Conversely, Lim et al. reported a cost-effectiveness analysis for LVAD use in HTx ineligible patients in the UK, showing an incremental cost-effectiveness ratio for LVAD vs. GMDT in inotrope-dependent hospitalized patients, whereas the cost-effectiveness ratio is significantly reduced for ambulatory patients that undergo LVAD implantation (34). However, it is worth underlying that the use of HM3 compared with other LVADs has significantly reduced the LVAD-related economic burden, as reported in the CLEAR-LVAD study, due to a significant reduction of LVAD-related complications (35). The cost-effectiveness analysis of both TEER and LVAD should be carried out in each country, owing to the significant differences in the health systems.

**Figure 2:**
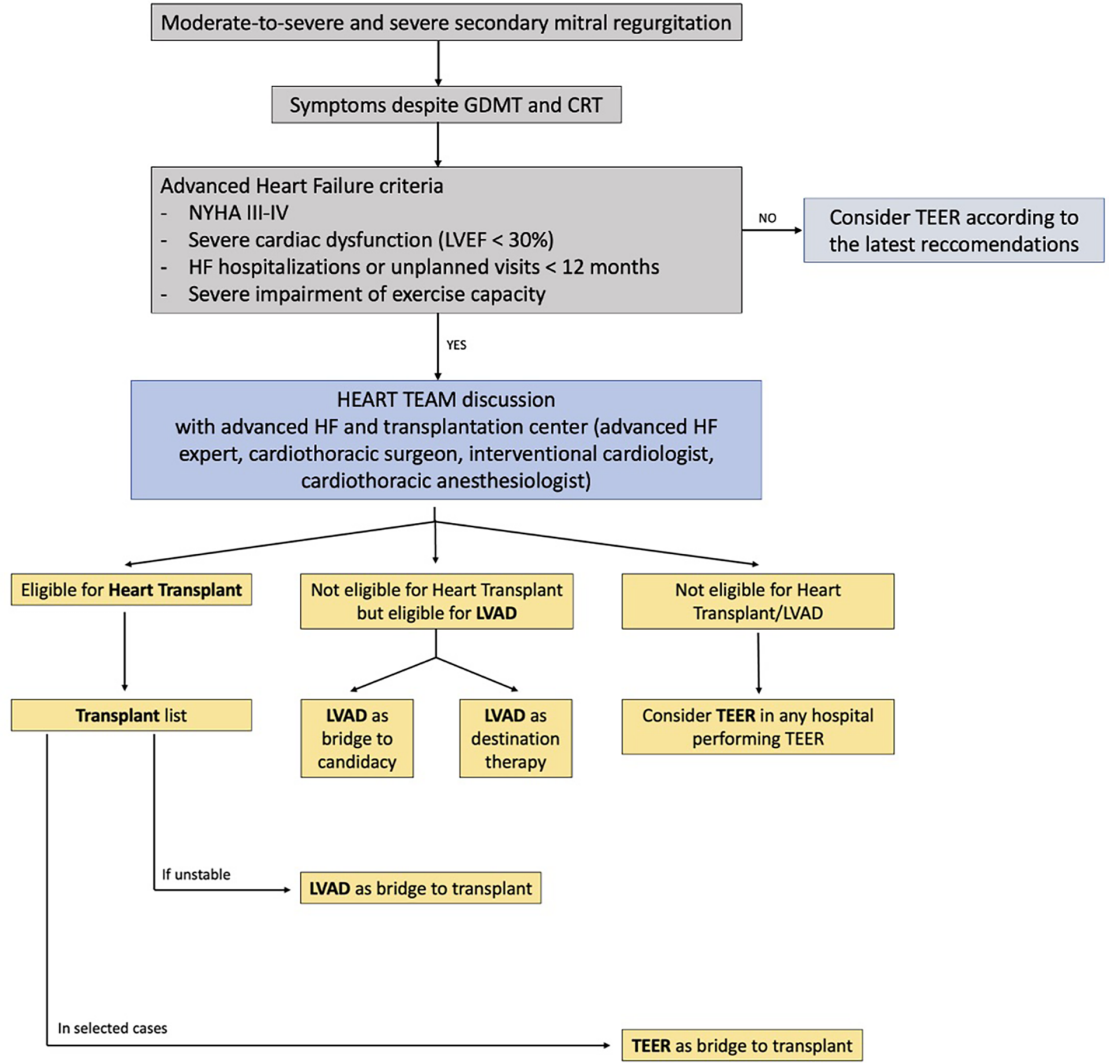
Flow chart for management of patients with moderate-to-severe secondary mitral regurgitation and advanced heart failure. The role of a multidisciplinary Heart Team, composed not only of interventional cardiologists but also of advanced heart failure experts and cardiothoracic surgeons, is essential to assess the optimal timing for interventional advanced heart failure therapy (heart transplantation, LVAD implantation, TEER).

A second explanation relies on the significantly higher number of cardiology departments where MitraClip can be performed compared with the relatively low number of hospitals in which LVAD can be implanted. A third explanation relies on the dimension of psychological acceptability of the treatment. If, on the one hand, TEER is significantly more accepted among both clinicians and patients, on the other hand, acceptance of LVAD is still very difficult to come by. Even HF specialists might see LVAD as a therapy that is too aggressive and definitive. In line with this thought, LVAD is considered as a therapeutic option only when the clinician has to face the decision whether or not start palliation, which is generally too late. In fact, in presence of severe right ventricular dysfunction, severely impaired renal or hepatic function, or severe cardiac cachexia, complications that could arise after long-standing advanced HF, LVAD could no longer represent a valid option, owing to the higher rate of post-implant complications that significantly decrease survival. Finally, another possible explanation is the lack of strong HF network between the hub and spoke centers as well as HF centers in which advanced HF strategies are available. In fact, one of the most frequent problems that these latter centers encounter is a late referral of advanced HF patients, which often precludes them from accessing these therapies, and which is probably associated with the scarce acceptability of LVAD as a therapeutic option. However, if the COAPT trial reported that TEER is a valid strategy to pursue in patients with SMR to obtain a clinical and survival benefit, it has to be stressed that in this trial patients with advanced HF were excluded. In the device group of both MITRA-FR trial and COAPT study, around 4% of patients had to undergo mechanical circulatory or Htx. On the other hand if we analyze the MitraBridge Registry, it appears that although it is true that TEER is safe in a sicker population such as patients on the HTx waiting list, almost 24% of the patients required urgent HTx or LVAD implant after the procedure. This consideration should underline the importance of a careful and thorough evaluation in patients presenting with refractory HF symptoms and significant MR, since TEER should not preclude them from being evaluated for HTx or LVAD implant but should be complimentary to it in selected cases (Figure 2). In this direction, it would be useful to set a country-specific registry of all patients that undergo TEER for SMR, to identify the subset of patients that might be classified with aHF and how many of them are actually referred to an HTX/LVAD center before or after TEER. Furthermore, future research should focus on the identification of HF patients in which TEER is associated with a high risk of urgent LVAD/HTx to avoid futile and potentially harmful procedures as well as to avoid late referral for advanced therapies. Several case series and small studies have reported that after TEER, LVAD implant is feasible and safe with no perisurgical complications related to the previous procedure (36–38). However, one aspect has to be factored in which is the number of implanted clips since multiple clips implantation might lead to a restrictive physiology that might preclude LVAD implant. Analyzing one case series of six patients, if it is true that no patients had surgical complication during LVAD implant related to the previous MitraClip procedure, it is also true that all patients underwent LVAD implant within almost a year from TEER (38). Furthermore, even though TEER successfully reduced the severity of MR, neither the echocardiographic parameters nor the hemodynamics improved significantly. This evidence points out that in a population of aHF patients, HF progresses despite the degree of MR or TEER procedure. Kreusser et al. showed in a retrospective study that despite the LVAD implant being safe and feasible after TEER, the use of TEER in patients with aHF with severe SMR might only delay LVAD implantation with potentially negative effects on long-term outcomes (39). In fact, the group of patients that underwent LVAD implantation after MitraClip, compared with patients that directly received LVAD, had a higher incidence of right ventricular (RV) failure and the need of RV assist device post-LVAD with worse outcome, even though this difference did not reach statistical significance (39). Finally, based on the recently released results from the MOMENTUM 3 investigation, LVAD implant significantly reduced the incidence as well as the severity of preimplant MR, from an incidence of 43.5%–6.2% after LVAD (40). Furthermore, the presence of MR before LVAD or during follow-up did not have a prognostic impact on this population (40).

**Figure 3:**
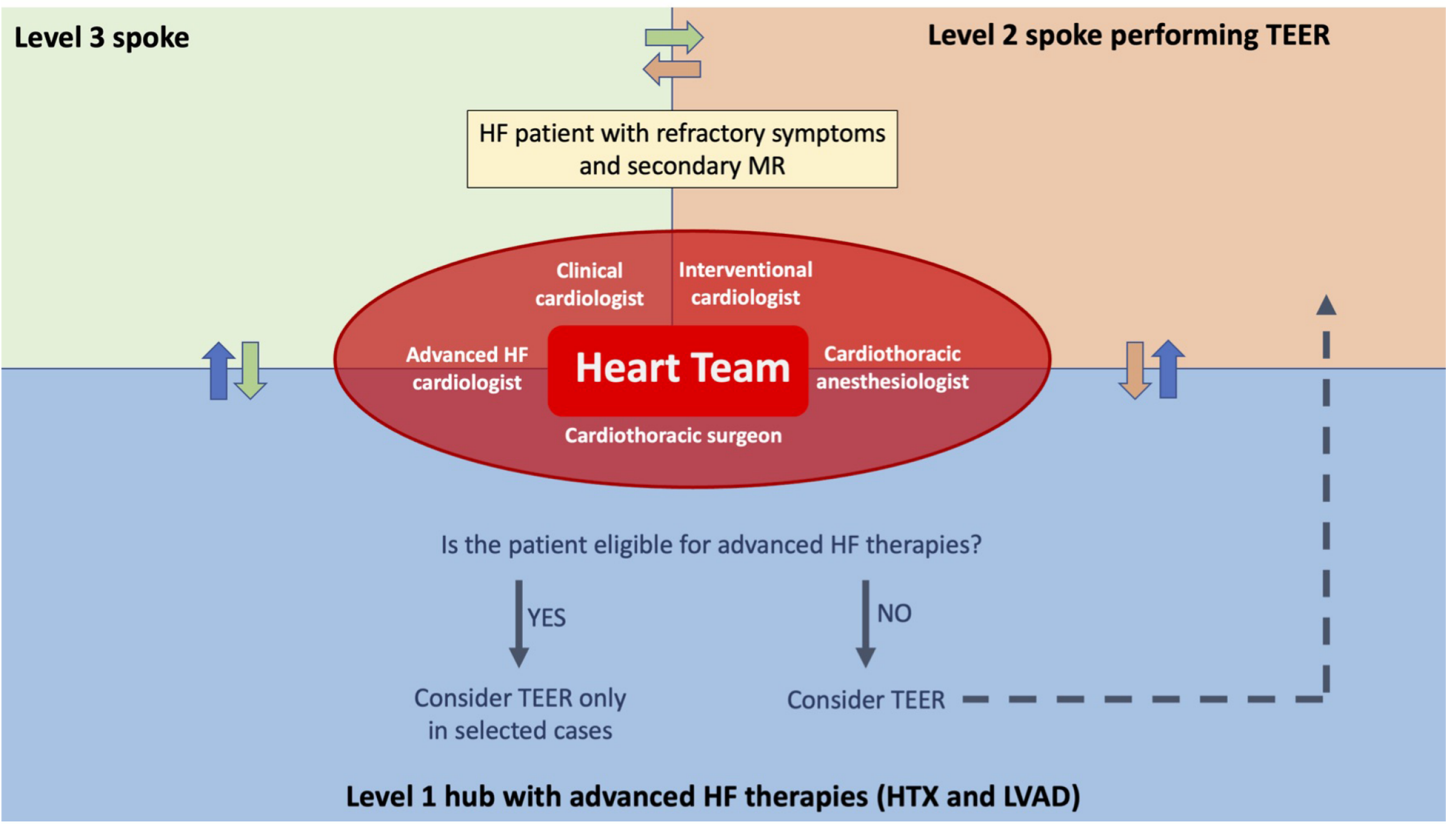
Advanced heart failure network for patients with significant secondary mitral regurgitation. An advanced HF network between hospitals is essential to avoid patients’ late referral for advanced HF therapies and to avoid futile procedures. With that aim, when a patient presents refractory HF symptoms and a significant secondary mitral regurgitation, discussion in the Heart Team, including interventional cardiologists and advanced HF cardiologists, is needed to define the best therapeutic option for that patient. In this way, if the patient is eligible for advanced therapies, such as HTx or LVAD, it is possible to proceed with a preliminary evaluation and, in selected cases, the patient could still undergo TEER if it is not judged futile. This is particularly important, on the one hand, for being able to perform HTx or LVAD implantation in a more urgent setting if TEER gets complicated and, on the other hand, to exclude patients that could hardly benefit from TEER and instead proceed with an LVAD implant and/or HTx listing. Finally, TEER can be performed in the tertiary center or in hospitals performing TEER, according to the patient's perioperative risk and to the interventional cardiologists’ preference.

Based on these premises, we believe that TEER might still be a valuable therapeutic option for patients with HF symptoms and SMR, even though it is essential to keep in mind that mortality remains high, especially in NYHA class IV patients. For this reason, patients that might be classified with aHF that are being considered for TEER should be discussed in the Heart Team, not only by the general cardiologist and interventional cardiologist but also a HF specialist in contact with a center in which HTx and/or LVAD are available options (Figure 3). This does not necessarily imply immediate LVAD implantation over TEER, but as stated above, it might improve patients’ selection and sometimes avoid futile procedures. Furthermore, it reduces the chances of losing patients that still underwent TEER at follow-up, since they were already notified to the HTx/LVAD center, facilitating the communications between hospitals and practitioners and therefore immediately intercepting initial signs of worsening HF.

Regarding the possible limitation of this review, there might have been a possible selection bias for not including smaller studies or registries in the discussion, since we considered the main available trials on LVADs and TEER. Furthermore, there are no studies directly comparing the use of LVAD and TEER, which might have shed further light on this topic, even though they are not practically realizable.

## Conclusions

Patients with aHF and moderate-to-severe or severe SMR, who are potentially eligible for HTx or LVAD, should be discussed in multidisciplinary Heart Teams, which should include aHF experts as well as interventional cardiologists, to avoid futile interventional procedures, define optimal timing for more advanced therapies, and carefully follow up with those patients that undergo TEER to avoid their future preclusion from HTx/LVAD resulting from late referral. To achieve this goal, it is essential to create a strong HF network between spoke, hub, and hospitals with HTx and LVAD.
